# Tumor visualization and fluorescence angiography with indocyanine green (ICG) in laparoscopic and robotic hepatobiliary surgery – valuation of early adopters from Germany

**DOI:** 10.1515/iss-2020-0019

**Published:** 2021-04-22

**Authors:** Mareike Franz, Jörg Arend, Stefanie Wolff, Aristotelis Perrakis, Mirhasan Rahimli, Victor-Radu Negrini, Jessica Stockheim, Eric Lorenz, Roland Croner

**Affiliations:** Department of General-, Visceral-, Vascular-, and Transplant-Surgery, University Hospital Magdeburg, Magdeburg, Germany

**Keywords:** CCC, CRLM indocyanine green, HCC, ICG, laparoscopic surgery, robotic surgery

## Abstract

**Objectives:**

Indocyanine green (ICG) is a fluorescent dye which was initially used for liver functional assessment. Moreover, it is of value for intraoperative visualization of liver segments and bile ducts or primary and secondary liver tumors. Especially in minimally invasive liver surgery, this is essential to enhance the precision of anatomical guided surgery and oncological quality. As early adopters of ICG implementation into laparoscopic and robotic-assisted liver surgery in Germany, we summarize the current recommendations and share our experiences.

**Methods:**

Actual strategies for ICG application in minimally invasive liver surgery were evaluated and summarized during a review of the literature. Experiences in patients who underwent laparoscopic or robotic-assisted liver surgery with intraoperative ICG staining between 2018 and 2020 from the Magdeburg registry for minimally invasive liver surgery (MD-MILS) were evaluated and the data were analyzed retrospectively.

**Results:**

ICG can be used to identify anatomical liver segments by fluorescence angiography via direct or indirect tissue staining. Fluorescence cholangiography visualizes the intra- and extrahepatic bile ducts. Primary and secondary liver tumors can be identified with a sensitivity of 69–100%. For this 0.5 mg/kg body weight ICG must be applicated intravenously 2–14 days prior to surgery. Within the MD-MILS we identified 18 patients which received ICG for intraoperative tumor staining of hepatocellular carcinoma (HCC), cholangiocarcinoma, peritoneal HCC metastases, adenoma, or colorectal liver metastases. The sensitivity for tumor staining was 100%. In 27.8% additional liver tumors were identified by ICG fluorescence. In 39% a false positive signal could be detected. This occurred mainly in cirrhotic livers.

**Conclusions:**

ICG staining is a simple and useful tool to assess individual hepatic anatomy or to detect tumors during minimally invasive liver surgery. It may enhance surgical precision and improve oncological quality. False-positive detection rates of liver tumors can be reduced by respecting the tumor entity and liver functional impairments.

## Background

**Figure 1:**
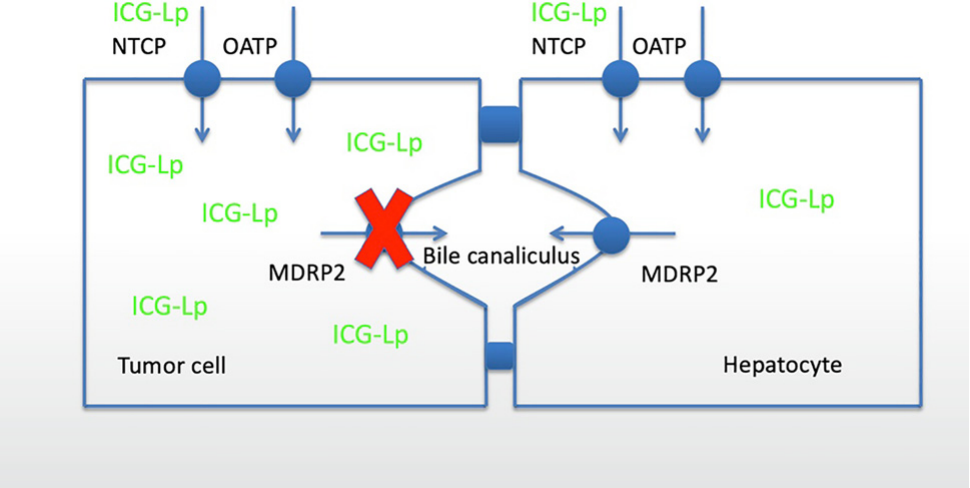
Cellular mechanisms of ICG uptake and secretion in liver tumors and healthy hepatocytes: ICG bound to lipoprotein is uptaken by hepatocytes and tumor cells via NTCP and OATP channels. Excretion is impaired in tumor cells whereas the ICG is excreted into the bile canaliculus via MDRP2 by hepatocytes.

Indocyanine green (ICG) is a fluorescent dye which gained acceptance in many different clinical fields since its admission by the U.S. Food and Drug Administration (FDA) in 1954. The absorption spectrum of ICG is in the near-infrared light (NIR). It emits protein-bound maximum fluorescence at about 840 nm when stimulated by near-infrared light [1], [2], [3]. After intravenous injection ICG binds to plasma proteins, especially albumin and alpha-1/beta-lipoproteins which are taken up by hepatocytes via organic anions-transporting polypeptides (OATP) and sodium taurocholate co-transporting polypeptides (NTCP). Finally, ICG is secreted unmetabolized into the bile via multidrug resistance-associated proteins 2 (MDRP2) [4], [5], [6] (Figure 1). Very few reports about adverse reactions or possible toxicity for ICG exist. Exceeding the recommended maximum dose of 2 mg/kg bodyweight a low frequency of adverse reactions including isolated anaphylaxis or mortality could be observed [6]. Adverse reactions with a dose of about 0.5 mg/kg bodyweight are reported with a frequency of 0.003% [7].

For the reason that ICG does not undergo the enterohepatic circulation, the ICG retention rate after 15 min of application (ICG R15) can be used to evaluate the liver function. The ICG R15 became a common dynamic preoperative instrument to evaluate the hepatic functional reserve before liver resection [1, 8, 9]. Currently, there is an increasing pre- and intraoperative implementation of ICG in hepatobiliary surgery for various settings.

Most of the studies concerning ICG in hepatobiliary surgery are from the east Asian region, mainly from Japan. Aoki et al. used ICG in 2008 to identify segments and sub-segments during anatomical liver resection [10]. It was observed that ICG can stain liver tumors and hepatic metastases [9, 10]. This finding became of interest for the resection of primary and secondary liver malignancies. With the development of specialized intraoperative imaging techniques in the near-infrared an increasing use of ICG in minimally invasive surgery was feasible. The visualization of extrahepatic bile ducts, e.g., as part of laparoscopic or robotic cholecystectomies or the detection of intraoperative bile duct leakages as so-called fluorescence-cholangiography, is an established scope for the use of ICG [11], [12], [13]. Furthermore, in minimally invasive tumor resection of the liver ICG may be useful to increase the intraoperative precision [14, 15]. Even though the use of ICG is established for hepatic surgery in several regions worldwide, in Germany it is used less so far. As an early adopter of minimally-invasive liver surgery in Germany, we here share our experience with ICG during laparoscopic and robotic liver resections.

## Fluorescence-guided liver mapping with ICG

**Figure 2:**
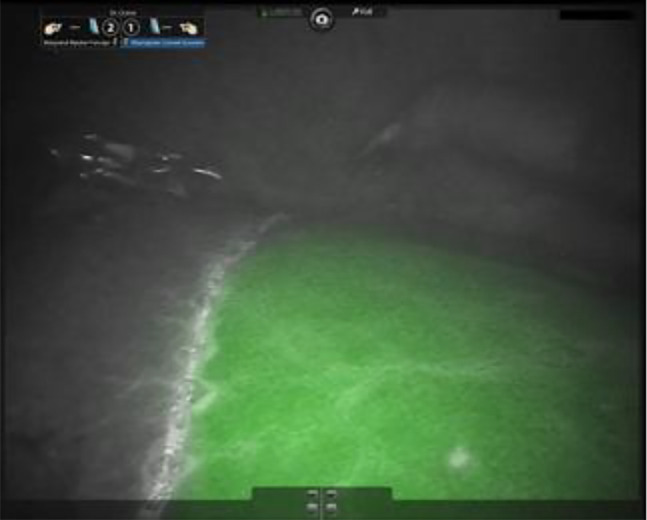
ICG staining of the left liver with intraoperative fluorescence angiography during robot-assisted liver resection, after ligation of the right hepatic artery and right portal vein branch. The demarcation line between the perfused left and nonperfused right liver becomes evident and enables precise anatomic right hemi-hepatectomy.

**Figure 3:**
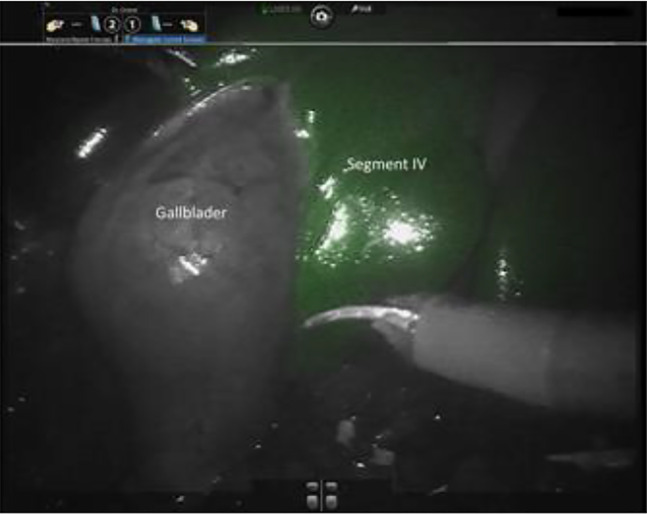
Intraoperative positive IGC staining of liver segment IV during robot-assisted liver surgery.

Residual nonperfused hepatic tissue after liver surgery increases postoperative morbidity. To prevent this reason for postoperative morbidity the liver segments have to be identified clearly during the operation. The visualization of these segments with ICG during intraoperative fluorescence angiography enables an anatomical resection [16] (Figures 2 and 3). Individual variations of liver anatomy can be identified and respected. Hereby, the maximum amount of liver capacity is preserved without neglecting anatomical principles [17, 18]. There is some evidence that the oncological outcome after anatomical vs. atypical liver resections is better for some tumor entities [19]. Therefore, the borders of the liver segments which can be variable have to be encountered. One possibility to identify the segments intraoperatively is a demarcation of the segment after selective interruption of the segmental blood supply. In open surgery, the demarcation is generally easy to identify whereas this can be challenging in a minimally invasive setting [19].

A better way to identify liver segments during minimally invasive liver surgery is a positive ICG staining [1]. Hereby, 5 mg of ICG is applicated via an ultrasound-guided puncture of the portal branch of the liver segment. Then the segment can be displayed with a photodynamic eye (PDE) fluorescence camera [20] (Figure 3). This procedure can be challenging in minimally invasive surgery and requires very good skills in handling and interpretation of the intraoperative ultrasound [21]. Another technique favors the clipping of the portal pedicle which supplies the anatomical segment intended for resection with blood. Then ICG is injected intravenously and the segment demarcates while the surrounding tissue is shining in green (negative staining). For the minimally invasive approach, this method is less complex compared to the positive staining via portal vein injection [21, 22].

But these techniques have some limitations. Urade et al. evaluated anatomical laparoscopic liver resections with intravenous ICG application. Sometimes the intersegmental borders could not be clearly identified by fluorescence imaging. As one reason for this finding portal venous shunts were identified [19, 22]. Furthermore, once ICG is injected another injection is not possible immediately, because the fluorescence signal of the segment that is intended for resection stays for a few hours [21].

An alternative to ICG staining is the liver segment identification using intraoperative contrast-enhanced ultrasound (CEUS) [23, 24]. But in the case of portal hypertension e.g., in patients with liver cirrhosis the sensitivity of CEUS is limited [24]. In addition, the intraoperative visualization of segments with ICG-fluorescence is not limited to patients with cirrhotic livers.

## Fluorescence cholangiography with ICG

In 1996, the procedure of fluorescence-cholangiography was used for the first time and offered an opportunity to replace the traditional method via X-ray [23].

The method gained importance due to its simplicity of perioperative handling [25]. Ishizawa et al. recommended an intravenous injection of 2.5 mg ICG 30 min prior to surgery [26]. The application can be performed intravenously or intrabiliary, e.g., into the cystic duct [27].

**Figure 4:**
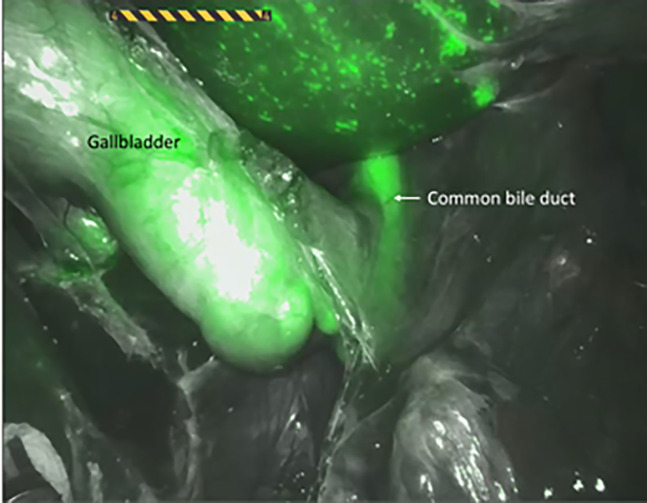
Fluorescence cholangiography during robot-assisted liver surgery. The common bile duct and the gallbladder are stained in green.

During laparoscopic cholecystectomies, the cystic duct can be visualized without preparation of the Calot’s triangle [28] (Figure 4). Small bile ducts and accessory gallbladder ducts as well as intra- or extrahepatic bile ducts can be identified sufficiently [29].

The visualization of extrahepatic bile ducts is important during cholecystectomies to prevent morbidity. Biliary leakages are a rare but severe complication [30]. They occur mainly during complex procedures for inflammatory gallbladders. Some studies report a significantly faster and safer identification of biliary structures by using ICG fluorescence compared to conventional minimal-invasive techniques [27, 31].

After liver resections, biliary fistulas can occur. Kaibori et al. [32] divided 102 patients into two groups to identify biliary leaks intraoperatively. One group was examined for biliary leakages with ICG fluorescence and the other group without fluorescence. Eight weeks after surgery the group without fluorescence control had significantly higher rates of postoperative complications and 10% biliary fistulas vs. 0% in the fluorescence group. These findings demonstrate the value of intraoperative ICG control for biliary leaks after partial liver resection.

## Liver tumor visualization with ICG

The crucial goal of every oncological liver resection is the complete removal of the tumor with adequate safety distance and at the same time the preservation of liver function by preserving as much healthy tissue as possible. An intraoperative tumor visualization can support this aim. Especially in minimally invasive liver surgery, the intraoperative ultrasound is essential to identify the borders of liver tumors to achieve sufficient resection margins [33, 34]. The precision especially in cirrhotic livers can be increased by adding ICG fluorescence to identify the tumor. In 2009 after preoperative intravenous injection of ICG as a part of liver functional testing fluorescence shining of HCCs and colorectal metastases could be visualized for the first time [24, 30, 35].

The mechanism of HCC ICG fluorescence was elucidated with immune-histochemical staining and gene expression analysis [36].

**Figure 5:**
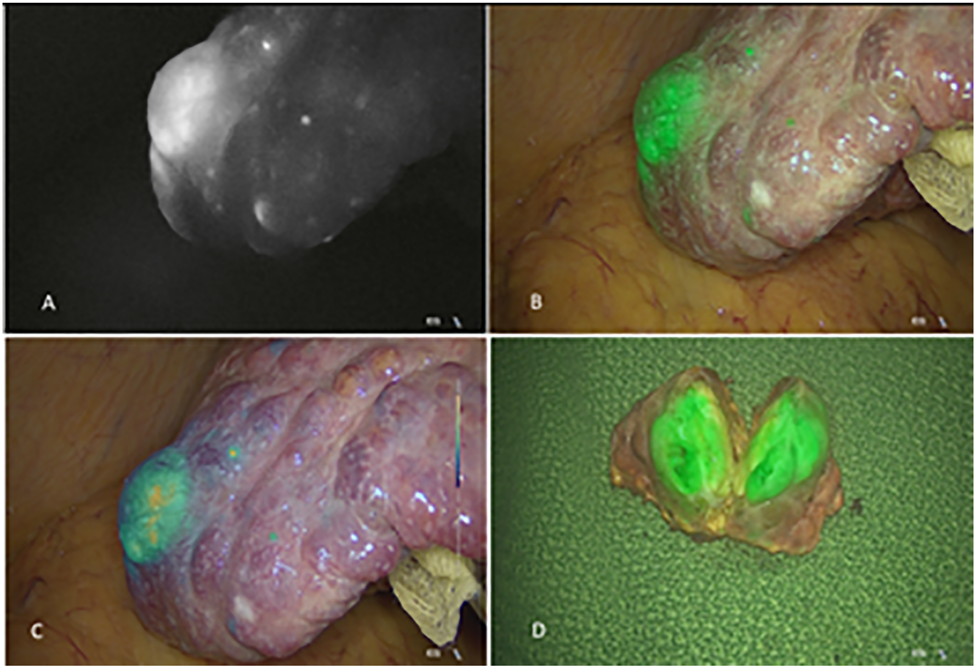
Intraoperative staining of hepatocellular carcinoma during laparoscopic liver resection. (A) Monochrome mode, (B) Homogenous fluorescence pattern indicating a well-differentiated HCC, (C) Intensity mapping, (D) Resected specimen which was divided to control the resection margins.

In well-differentiated HCC cells, there is a similar or even higher expression of OATP and natrium–taurocholate co-transporting polypeptides (NTCP) for admission of lipoprotein-bound ICG [1, 21, 37, 38]. In contrast to healthy hepatocytes the bile excretion is disturbed [1, 21, 38, 39] (Figure 1). This mechanism results in a retention of ICG after preoperative intravenous injection in tumorous tissue and is consequently a typical homogenous fluorescence pattern for well-differentiated HCCs [11, 39] (Figure 5). In poor differentiated HCCs the portal uptake by OATP and NTCP is limited, at the same time the biliary excretion is disturbed as well, therefore an inhomogeneous, or even rim-type-fluorescence pattern results [39].

**Figure 6:**
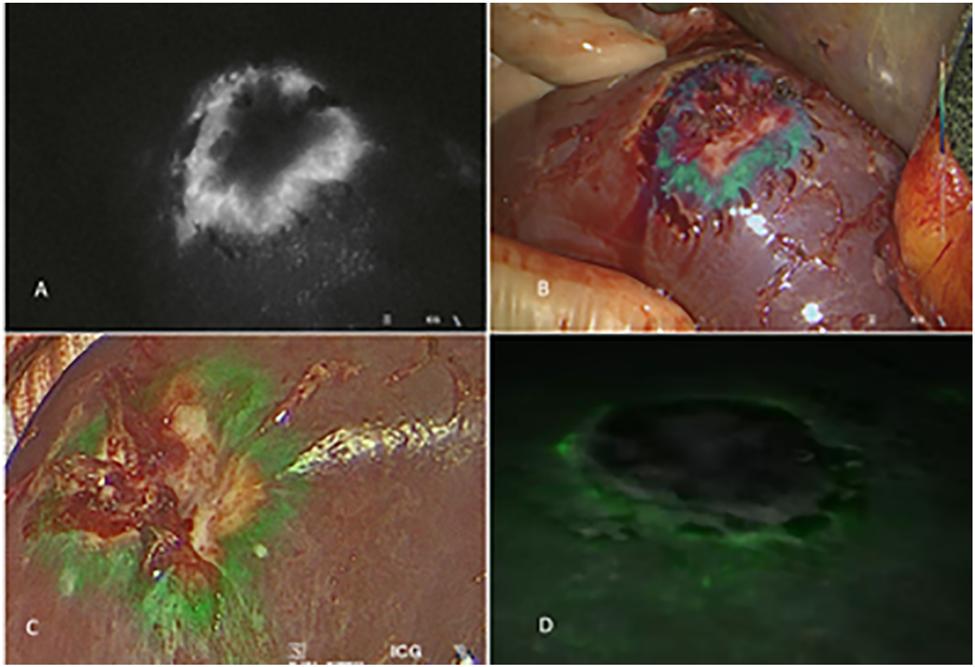
Intraoperative visualization of colorectal liver metastases during liver surgery. The typical rim-type fluorescence pattern can be identified. (A) Monochrome mode, (B) Intensity mapping, (C) Laparoscopic approach, (D) Robotic approach.

**Figure 7:**
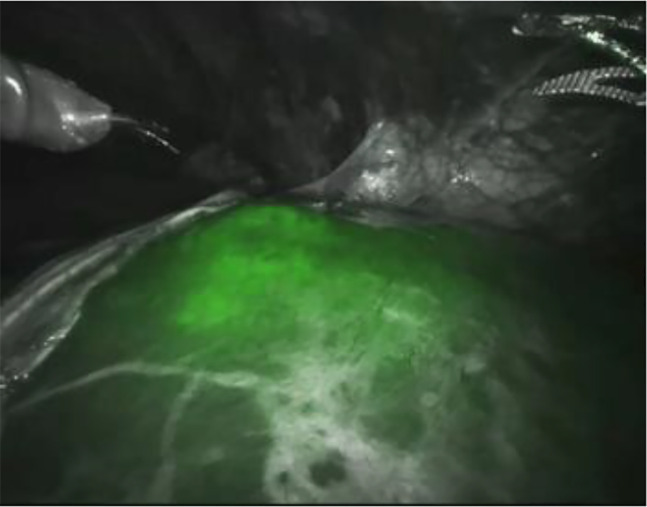
Intraoperative visualization of an intrahepatic cholangiocellular carcinoma (CCC)-visualization of the surrounding areas of cholestasis.

CRLM is accessible for ICG visualization as well. A surrounding fringe of immature hepatocytes with reduced bile excretion capacity shows a rim-type fluorescence pattern [11, 39] (Figure 6). This is a typical feature for this type of intrahepatic secondary malignancies. In cholangiocellular carcinomas (CCC) there is limited evidence for intraoperative ICG staining. In this tumor type, not the tumor itself but the surrounding areas of cholestasis show green fluorescence [40, 41] (Figure 7). Therefore, the tumor borders cannot be identified by ICG staining only which makes the use of an intraoperative ultrasound indispensable.

For intraoperative staining of liver tumors with ICG, an intravenous injection of 0.5 mg/kg bodyweight of ICG is recommended 2–14 days prior to surgery (Table 1). Recommendations for dose or time of injection regarding the tumor type are not standardized in the literature so far [24, 36, 41].

**Table 1:** References [24, 36, 41–43] dose, recommended timepoint of systemic, preoperative injection, limitations, and detection rates of indocyanine green (IGC) for hepatocellular carcinomas (HCC), colorectal (CRC) liver metastases, and cholangiocellular carcinomas (CCC).

	HCC	CRC metastases	CCC
Dose	0.5 mg/kg KG	0.5 mg/kg KG	0.5 mg/kg KG
Timepoint of intravenous injection	2–14 days preoperative	2–14 days preoperative	2–14 days preoperative
Limitations	Depths of visualization, False-positive signals due to bad liver function, depending on studies 8–40% false-positive results	Depths of visualization, false-positive signals due to bad liver function, Limited assessability of bad-differentiated HCCs and metastases	Depths of visualization, false-positive signals due to bad liver function, indirect visualization by surrounding cholestasis
Detection rate	70–100%	69–100%	100% (less data)

The sensitivity of fluorescence-guided visualization of primary and secondary liver tumors was reported between 0 and 100% in the literature [24, 36, 42, 43]. Especially in minimally invasive hepatobiliary surgery that lacks the palpation of the tumor as a haptic feedback, fluorescence identification seems to be helpful [36, 44]. The combination of intraoperative ultrasound and ICG staining enhances the precision for tumor detection and clear resection margins. Zhang et al. [42] reported that 12 tumor lesions in eight patients, which have not been identified in preoperative standard imaging, could be detected by intraoperative ICG fluorescence. The smallest lesion had a diameter of about 2 mm in this study.

**Figure 8:**
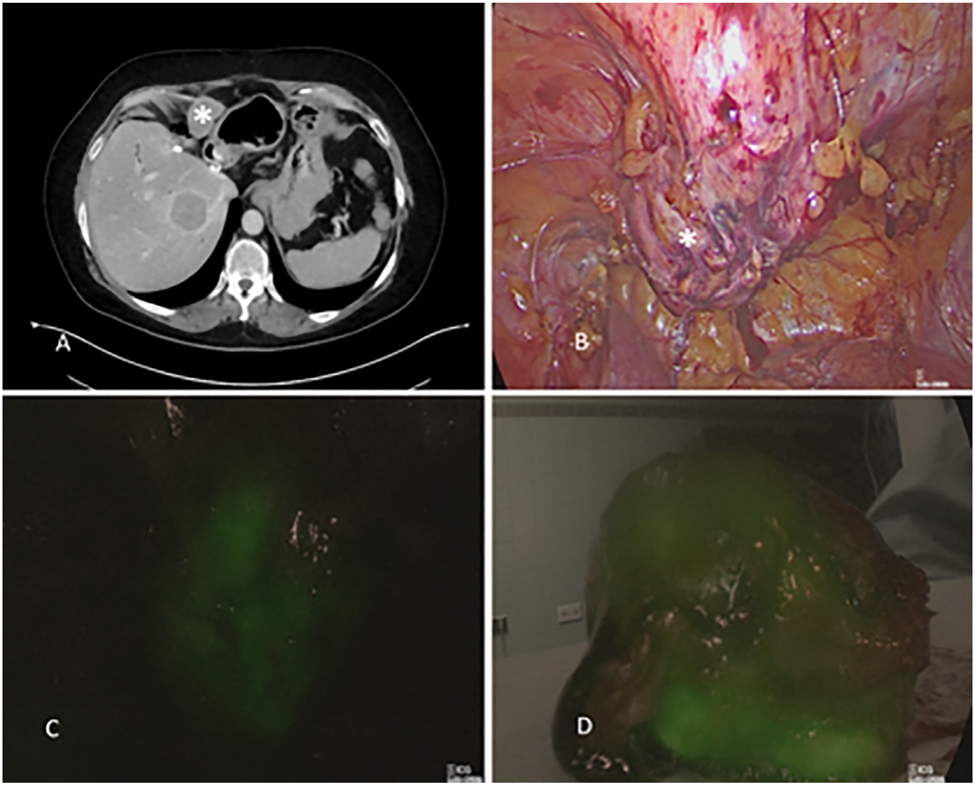
Identification of peritoneal metastasis from hepatocellular carcinoma (HCC). *Indicates the HCC metastasis, (A) CT scan, (B) Laparoscopic identification of the HCC metastasis, (C) ICG fluorescence of HCC metastasis during laparoscopy, (D) ICG fluorescence of the removed and divided HCC metastasis.

In cases of advanced HCC extrahepatic metastases can occur, in adrenals, lungs, lymph nodes, peritoneum, skeletal metastases, etc. In 2013 Satou et al. [45] found fluorescence signals in already known extrahepatic metastases as well as in not yet detected metastases after preoperative ICG injection in patients with HCC. These reports are in concordance with our experiences where we were able to identify the peritoneal spread of HCC during laparoscopic peritoneal exploration (Figure 8)

**Figure 9:**
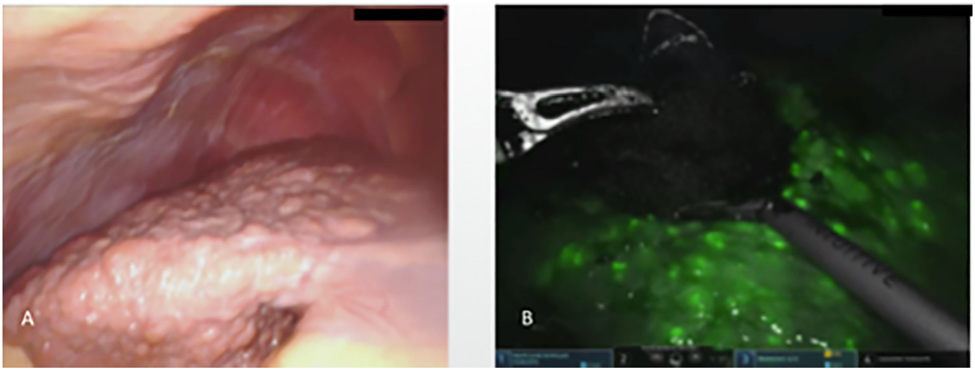
(A) Cirrhotic liver identified during robot-assisted liver surgery, (B) False-positive ICG fluorescence of regenerative nodules in the cirrhotic liver intraoperative visualization of an intrahepatic cholangiocellular carcinoma (CCC)-visualization of the surrounding areas of cholestasis

But there are some limitations of the fluorescence-visualization of tumors with ICG. Maximum tissue penetration of 5–10 mm of the fluorescent light into the liver tissue is described. Therefore, only subcapsular lesions can be visualized [30, 46]. A study by Kudo et al. elucidated that tumors 8 mm or deeper under the liver surface cannot be identified by ICG fluorescence anymore [44]. False-positive fluorescence signals are another problem with ICG fluorescence (Figure 9). In patients with impaired liver function, the capacity of bile excretion is reduced [46, 47]. Eight percent of false-positive signals were detected in a series of 63 HCC patients by Ishizawa et al. [35]. In other series´ false-positive signals were reported in up to 40% [47]. By now a standardized recommendation about the time of the preoperative ICG injection does not exist, however, a longer interval before the operation seems to reduce the rate of false-positive signals in patients with impaired liver function [1, 48].

## Preliminary own experiences of ICG liver tumor staining

Since 12/2018 we use ICG pre- or intraoperative as part of laparoscopic or robot-assisted hepatobiliary resections. 18 patients received ICG prior to surgery with the intention of tumor visualization (Table 2). 61% (11/18) underwent robotic-assisted resection with the Da Vinci Xi System (Intuitive Inc., Santa Clara, USA) using the firefly-integrated fluorescence capability and 39% (7/18) patients underwent laparoscopic resection using an endoscope with near-infrared indocyanine green (NIR/ICG) system (Karl Storz, Tuttlingen, Germany). Nine male and nine female patients were included in our cohort with a mean age of 77 years (range 47–86 years) and a mean American Society of Anesthesiologists (ASA) score of 3 (2.67). We included patients with any type of liver tumor and in one case peritoneal spread (one singular metastasis) of HCC. Fifty percent of our patients (9/18) suffered from HCC, 22% (4/18) from CRLM, 11% (2/18) had intrahepatic cholangiocellular carcinoma (CCC) and 6% (1/18) adenoma, a peritoneal manifestation of HCC and a rare mix-type tumor entity HCC/CCC with stem-cell characteristics. All patients signed written consent. Exclusion criteria for application of ICG were iodine allergy, severe renal function impairments, hyperthyreosis, and a tendency for anaphylaxis.

**Table 2:** Demographic and perioperative data of 18 patients from MD-MILS who underwent minimally invasive liver resection and were applicated ICG for intraoperative tumor visualization prior to surgery.

	
Total patients, n	18
Age at surgery, median (range)	77 (47–86)
**Gender, n (%)**	
Female	50 (9)
Male	50 (9)
BMI, median (range)	27 (20–34)
**ASA classification, n (%)**	
1	0
2	33 (6)
3	67 (12)
4	0
Liver cirrhosis/-fibrosis, n (%)	33 (6)
Previous liver surgery, n (%)	11 (2)
LiMax liver function assessment, median (range), standard deviation, µg/kg/h	363 (175–657), 122
**Classification of surgical complications- Clavien Dindo, n (%)**	
0	67 (12)
1	11 (2)
2	11 (2)
3a	11 (2)
3b	0
4a	0
4b	0
5	0
Operation time minutes, median (range)	257.2 (72–515)
Hospital stay, median (range)	10.6 (4–21)
Major resection (3 or more segments), n (%)	44 (8)
Minor resection (2 or less segments), n (%)	56 (10)

We inject ICG in a dose of 0.5 mg/kg body weight, 2–10 days prior to surgery for intraoperative tumor visualization. No adverse reactions occurred in our cohort. Our time of injection depended on the individual liver function of the patient (Table 1). We include the preoperative LiMax^®^-liver function assessment as standard procedure in our cohort. A mean LiMax^®^ of 363 microgram/kg body weight (range 175–657; standard deviation 122) was identified. Risk factors for liver dysfunction such as chronic alcohol abuse, diabetes, chemotherapies, and laboratory liver function parameters were included in our decision making for the time of preoperative ICG injection. The mean preoperative time of injection in our cohort was four days (range 1–10 days).

In 27.8% of the cases, we saw intraoperative fluorescence signals of pre- and intraoperative nondetectable primary liver tumors or extrahepatic metastases that led to a change of the therapeutic strategy, e.g., extension of the resection, or termination of the operation. In every case, we saw a tumor fluorescence signal of the already known lesions, so in our collective, we found a sensitivity of the intraoperative tumor presentation of 100%. As already mentioned a maximum tissue penetration of 5–10 mm for fluorescence light is described [30, 46]. For this reason, we used ICG fluorescence as margin control in deep intrahepatic lesions. In 100% tumor visualization of our cohort, the R0 resection rate was within that range.

Nevertheless, the false-positive signal of 39% in our patients indeed shows a limitation of this visualization method. We identified false-positive signals in regenerate nodules as part of cirrhotic deformed liver parenchyma, small cysts, von- Meyenburg-complexes, or focal parenchyma changes. We considered that in most of our cases with an/the impaired liver function we chose the interval of ICG injection prior to surgery too short. We figured out that in cirrhotic livers, the nontumorous parenchyma needs 7–10 days to excrete ICG into the bile. Therefore, a more precise selection of the time period of injection prior to surgery may reduce the rate of false-positive fluorescence signals.

## Discussion

ICG-fluorescence offers a simple and safe method for visualization of liver tissue, bile ducts, and tumors in hepatobiliary surgery [24, 49]. In comparison to the East Asian area, there is no routine use of ICG in hepatobiliary surgery in Europe and North America, despite its simple and safe application [24]. Ali Majlesara et al. [24] considered the nonroutine implementation of ICG for preoperative liver function assessment as one possible reason for that finding. Nevertheless, during the increasing implementation of minimally invasive liver surgery in western countries ICG gains more and more recognition. For the reason that haptic feedback for tumor identification is missing during these procedures, other tools become mandatory to reach sufficient oncological results. The intraoperative ultrasound and even the contrast-enhanced technique can facilitate this demand but ICG can add more precision. And this not only in liver tumor removal but also in anatomical liver resections by identifying individual segmental anatomy of the liver.

Currently, ICG is investigated not only for its diagnostic reasons but also as a therapeutic agent in liver tumors. The characteristic of ICG to accumulate in HCC cells is a topic that Inagaki et al. focused on in an experimental study. They examined ICG-conjugated chemotherapeutics, Gemcitabine, Doxorubicin, *in vitro* and *in vivo* in nude mice and found that ICG-conjugated Gemcitabine seems to be less toxic against normal cells and show better effects against tumor cells in contrast to unconjugated Gemcitabine [38]. This could be a possible future therapeutic innovation. Another possible future scenario could be that specific tissue- and tumor-specific antibodies could be used that bind to fluorophores for intraoperative visualization of these structures. That could specifically define tumorous tissue and metastases during intraoperative imaging and lead to adaption of the intraoperative strategy [50].

In summary, we figured out that the use of ICG is safe and simple. Unfortunately, its implementation in hepatobiliary surgery in Europe and especially in Germany is still underrepresented. It is true that there is currently a relevant false positive rate for tumor identification but this could be specified by more precise ICG application regarding the liver function and tumor aspects in the future. The limited tissue penetration does not seem to be a relevant limitation as there is still a sensitivity of 70–100% reported in the literature which is in concordance of our experiences during intraoperative tumor visualization. Furthermore, ICG enables a real-time-margin control for deeper lesions [51]. The identification of liver segments and bile ducts enhances the quality of liver surgery and can be a sufficient tool to reduce morbidity in the future.
