# Supplementary material for: Tumor visualization and fluorescence angiography with indocyanine green (ICG) in laparoscopic and robotic hepatobiliary surgery – valuation of early adopters from Germany
Source: Innov Surg Sci. 2021 Apr 22;6(2):59–66. doi: 10.1515/iss-2020-0019 (PMC8435269; doi:10.1515/iss-2020-0019)
Supplement: Supplementary file 1 [file iss-06-20200019-s001.pdf]

Reviewer Assessment

M. Franz et al.: Tumor visualization and fluorescence angiography with indocyanine green (ICG) in laparoscopic and robotic hepatobiliary surgery

Reviewers' Comments to Original Submission

Reviewer 1: anonymous

Date received: 8-Sep-2020
Reviewer recommendation: **Return to author for major modifications**
Reviewer overall scoring: **Medium**

Assessment Form scores: 5 = High/Yes; 3 = Medium/Adequate; 1 = Low

Is the subject area appropriate for the journal		4			
Does the title clearly reflect the paper's content?			3		
Does the abstract clearly reflect the paper's content			3		
Do the keywords clearly reflect the paper's content?			3		
Does the introduction present the problem clearly?			3		
Are the results/ conclusions justified?				2	
How comprehensive and up-to-date is the subject matter presented?					
How adequate is the data presentation?				2	
Are units and terminology used correctly?			3		
Is the number of cases adequate?				2	
Are the experimental methods/ clinical studies adequate?			3		
Is the length appropriate in relation to the content?			3		
Does the reader get new insights from the article?			3		
Please rate the practical significance.			3		
Please rate the accuracy of methods.				2	
Please rate the statistical evaluation and quality control.			3		
Please rate the appropriateness of the figures and tables.			3		
Please rate the appropriateness of the references.					1
Please evaluate the writing style and use of language.					1
Please judge the overall scientific quality of the manuscript.			3		
Are the methods used worthy of reproduction in greater deal?			No		
Would you be willing to review a revision of this manuscript?			No		

Comments to author: The paper describes a review of the literature on ICG in hepatobiliary surgery. The group also describes their own experiences in 18 cases of liver surgery in a time period of 18 months, from a variety of entities, while one case seems to be an extrahepatic metastasis.
The topic of ICG staining in liver surgery is of interest to the surgical community and I agree with the authors, that the implementation in Germany is rather slow. The authors describe the different aspects of the ICG technique and describe their own cohort.
The manuscript adds only little to what is already known about ICG but it is a more or less new area in the surgical community.

General comments:
The manuscript should be significantly edited by a native speaker.
The References are not well displayed, the style is far from the journals' requirements. The manuscripts cited are even hard to identify. A thorough check of the cited literature is impossible and inacceptable for a review article. This part needs significant editing.
Table 1: on which data/literature sources are the percentages of the detection rate based on?
There are two descriptions of Figure 9.
The authors should provide a table with the different use and different application of ICG (summary of the topics Flourescence-guided liver mapping, fluorescence cholangiography, visualization of liver tumors)
Specific: The maximum tissue penetration of only 5-10 mm is a major limitation of the method. This point should be worked out/discussed more in detail.
Authors´ own cohort:
Was ICG used in all laparoscopic/robotic liver resections? If not, what have been the selection criteria?
18 patients is a rather small cohort and enables a detailed description of the cases as a Table including detailed pre- intra- and postoperative Information
The Limax test is described as a preoperative standard. Liver function of the individual patients would be interesting, especially since the application of ICG was based on liver function as stated in the text.

In conclusion:
The paper refers to a new an interesting topic for liver surgeons. The information given in the paper is not new but it is a developing field and not yet entirely in the view of many surgeons.
The paper is submitted as original article but the authors only briefly report on their experience with 18 cases. So, in its current version the paper is rather an original than a review article.
The authors should re-write this paper and " Tumor visualization and fluorescence angiography with indocyanine green (ICG) in laparoscopic and robotic hepatobiliary surgery – experience with 18 cases and a review of the literature"
The paper needs a linguistic revision.

Reviewer 2: anonymous

Date received: 12-Oct-2020
Reviewer recommendation: **Return to author for major modifications**
Reviewer overall scoring: **Medium**

Assessment Form scores: 5 = High/Yes; 3 = Medium/Adequate; 1 = Low

Is the subject area appropriate for the journal		4			
Does the title clearly reflect the paper's content?		4			
Does the abstract clearly reflect the paper's content		4			
Do the keywords clearly reflect the paper's content?		4			
Does the introduction present the problem clearly?		4			
Are the results/ conclusions justified?				2	
How comprehensive and up-to-date is the subject matter presented?	5				

How adequate is the data presentation?					1
Are units and terminology used correctly?					
Is the number of cases adequate?			3		
Are the experimental methods/ clinical studies adequate?					1
Is the length appropriate in relation to the content?				2	
Does the reader get new insights from the article?		4			
Please rate the practical significance.		4			
Please rate the accuracy of methods.				2	
Please rate the statistical evaluation and quality control.					
Please rate the appropriateness of the figures and tables.			3		
Please rate the appropriateness of the references.					
Please evaluate the writing style and use of language.		4			
Please judge the overall scientific quality of the manuscript.				2	
Are the methods used worthy of reproduction in greater deal?					
Would you be willing to review a revision of this manuscript?	No				

Comments to author: The topic is very interesting and the paper is well written. But informations about their own cases are necessary. Which kind of resection? Lengths of procedures? Postoperative Outcome? Which system is used in laparoscopic cases? and so one.

Authors' Response to Reviewer Comments

Date received: 27-Oct-2020

Response to reviewer 1

General comments:

The manuscript should be significantly edited by a native speaker.

R: The manuscript was edited regarding the language as recommended

The References are not well displayed, the style is far from the journals' requirements. The manuscripts cited are even hard to identify. A thorough check of the cited literature is impossible and inacceptable for a review article. This part needs significant editing.

R: References were edited as recommended, the style was adopted to the manuscript

Table 1: on which data/literature sources are the percentages of the detection rate based on?

R: The references were added to table 1 one as requested.

There are two descriptions of Figure 9.

R: There is only one description of figure nine, the second one was deleted

The authors should provide a table with the different use and different application of ICG (summary of the topics Flourescence-guided liver mapping, fluorescence cholangiography, visualization of liver tumors)

R: A table with the different use and application of ICG is an interesting addition to the short overview we gave but we mainly wanted to show an overview over the topic and share our own experiences in tumor visualization therefore we did not focus on the different use of ICG - fluorescence in liver surgery

Specific: The maximum tissue penetration of only 5-10 mm is a major limitation of the method. This point should be worked out/discussed more in detail.

R: The maximum tissue penetration of 5-10 mm has not been a limitation in our own cohort by now. We saw a sensitivity of 100% in our cohort for tumor visualization. For deeper lesions we recommend the use of ICG for a real- time margin-control of the resection margins as stated in the article.

Authors´ own cohort:

Was ICG used in all laparoscopic/robotic liver resections? If not, what have been the selection criteria?

R: Exclusion criteria for ICG were included in the text : Exclusion criteria for application of ICG were iodine allergy, severe renal function impairments, hyperthyreosis and a tendency for anaphylaxis.

18 patients is a rather small cohort and enables a detailed description of the cases as a Table including detailed pre- intra- and postoperative Information

R: More information to the cohort and our own experiences were added as demanded

The Limax test is described as a preoperative standard. Liver function of the individual patients would be interesting, especially since the application of ICG was based on liver function as stated in the text.

R: Table 2 with information to the LiMax test results was added to the manuscript

In conclusion:

The paper refers to a new an interesting topic for liver surgeons. The information given in the paper is not new but it is a developing field and not yet entirely in the view of many surgeons.

The paper is submitted as original article but the authors only briefly report on their experience with 18 cases. So, in its current version the paper is rather an original than a review article.

The authors should re-write this paper and

“ Tumor visualization and fluorescence angiography with indocyanine green (ICG) in laparoscopic and robotic hepatobiliary surgery – experience with 18 cases and a review of the literature”

The paper needs a linguistic revision.

R: The title has been changed and the experiences with own cases were included in table 2. We agree that ICG is used more common internationally, but for the reason that ICG is a new tool in Germany regarding liver surgery and tumor visualization a cohort of 18 patients is worth it getting recognized.

Response to reviewer 2

The topic is very interesting and the paper is well written. But informations about their own cases are necessary. Which kind of resection? Lengths of procedures? Postoperative Outcome? Which system is used in laparoscopic cases? and so one.

R: Information to demographic and perioperative data has been added to the text as recommended and a table with further information (table 2) was attached.

Reviewers' Comments to Revised Submission

Reviewer 1: anonymous

Date received: 10-Jan-2021
Reviewer recommendation: **Return to author for minor modifications**
Reviewer overall scoring: **High**

Assessment Form scores: 5 = High/Yes; 3 = Medium/Adequate; 1 = Low

Is the subject area appropriate for the journal		4			
Does the title clearly reflect the paper's content?			3		
Does the abstract clearly reflect the paper's content			3		
Do the keywords clearly reflect the paper's content?		4			
Does the introduction present the problem clearly?			3		
Are the results/ conclusions justified?			3		
How comprehensive and up-to-date is the subject matter presented?				2	
How adequate is the data presentation?			3		
Are units and terminology used correctly?					
Is the number of cases adequate?				2	
Are the experimental methods/ clinical studies adequate?			3		
Is the length appropriate in relation to the content?			3		
Does the reader get new insights from the article?			3		
Please rate the practical significance.		4			
Please rate the accuracy of methods.			3		
Please rate the statistical evaluation and quality control.				2	
Please rate the appropriateness of the figures and tables.			3		
Please rate the appropriateness of the references.			3		
Please evaluate the writing style and use of language.			3		
Please judge the overall scientific quality of the manuscript.			3		

Are the methods used worthy of reproduction in greater deal?	No
Would you be willing to review a revision of this manuscript?	No

Comments to author: Thank you for the opportunity to review the manuscript after revision.
The authors have successfully corrected the errors in the general form of the manuscript and have edited it to the journals requirements. The language has been improved.
The references the data in table 1 is based on has been included, yet it is missing in the submitted version. This should be corrected before acceptance.
The own cohort of the authors is now well described with more details to the underlying disease, patients´ characteristics and postoperative outcome. The reflection of the experience of the authors with ICG gains more information now.
I suggest the acceptance of the manuscript after revision.

Reviewer 3: Gumbs, Andrew

Date received: 10-Dec-2020
Reviewer recommendation: **Accept in present form**
Reviewer overall scoring: **Excellent**

Assessment Form scores: 5 = High/Yes; 3 = Medium/Adequate; 1 = Low

Is the subject area appropriate for the journal	5				
Does the title clearly reflect the paper's content?	5				
Does the abstract clearly reflect the paper's content	5				
Do the keywords clearly reflect the paper's content?	5				
Does the introduction present the problem clearly?	5				
Are the results/ conclusions justified?					
How comprehensive and up-to-date is the subject matter presented?	5				
How adequate is the data presentation?	5				
Are units and terminology used correctly?	5				
Is the number of cases adequate?		4			
Are the experimental methods/ clinical studies adequate?	5				
Is the length appropriate in relation to the content?	5				
Does the reader get new insights from the article?		4			
Please rate the practical significance.	5				
Please rate the accuracy of methods.	5				
Please rate the statistical evaluation and quality control.	5				
Please rate the appropriateness of the figures and tables.	5				
Please rate the appropriateness of the references.		4			
Please evaluate the writing style and use of language.		4			
Please judge the overall scientific quality of the manuscript.	5				
Are the methods used worthy of reproduction in greater deal?	No				
Would you be willing to review a revision of this manuscript?	Yes				

Comments to author: This is an excellent article on a technique that needs more exposure in Europe and North America. The authors addressed both Reviewer's comments exceedingly well, I agree that the requested title change does not add anything to the manuscript and is not necessary.

Authors' Response to Reviewer Comments on revised manuscript

Date received: 10-Jan-2021

Response to reviewer 1

The references the data table 1 is based on has been included, yet it is missing in the submitted version. This should be corrected before acceptance.

R: Thank you for your comment. We added the references to the table.

Comments by the Editor-in-Chief to 2nd revised Submission

All reviewer concerns raised were addressed satisfactorily. The manuscript may be published in the present stage.
